# Antibiotic knowledge, attitudes and reported practice during pregnancy and six months after birth: a follow- up study in Lao PDR

**DOI:** 10.1186/s12884-022-05018-x

**Published:** 2022-09-12

**Authors:** Sengchanh Kounnavong, Weirong Yan, Amphoy Sihavong, Vanphanom Sychareun, Jaran Eriksen, Claudia Hanson, Kongmany Chaleunvong, Bounxou Keohavong, Manivanh Vongsouvath, Mayfong Mayxay, Annelie Brauner, Cecilia Stålsby Lundborg, Anna Machowska

**Affiliations:** 1 Lao Tropical and Public Health Institute, Ministry of Health, Vientiane, Lao PDR; 2 Department of Global Public Health, Karolinska Institutet, Stockholm, Sweden; 3 Vientiane Capital Health Department, Ministry of Health, Vientiane, Lao PDR; 4 Faculty of Public Health, University of Health Sciences, Vientiane, Lao PDR; 5 Department of Infectious Diseases/Venhalsan, Stockholm South General Hospital, Stockholm, Sweden; 6 Institute of Research and Education Development, University of Health Sciences, Vientiane, Lao PDR; 7 Food and Drug Department, Ministry of Health, Vientiane, Lao PDR; 8 Lao-Oxford-Mahosot Hospital-Welcome Trust Research Unit (LOMWRU), Microbiology Laboratory, Mahosot Hospital, Vientiane, Lao PDR; 9 Institute of Research and Education Development, UHS, Ministry of Health, Vientiane, Lao PDR; 10 Centre for Tropical Medicine and Global Health, University of Oxford, Oxford, UK; 11 Department of Microbiology, Tumor and Cell Biology, Karolinska Institutet, Stockholm, Sweden; 12 Division of Clinical Microbiology, Karolinska University Hospital, Stockholm, Sweden

**Keywords:** Antibiotic use, Pregnancy, Childhood illness, Lao PDR, Antibiotic resistance

## Abstract

**Background:**

Antibiotics are important medicines to prevent maternal and child morbidity and mortality. Women’s knowledge and attitudes towards antibiotic use influence their practice. When they become mothers, this may be mirrored in the use of antibiotics for their newborn children. The current study aimed to assess knowledge, attitudes, and reported practice of pregnant women regarding antibiotic use and antibiotic resistance as well as their approach towards antibiotic use for their newborn babies.

**Methods:**

This was a follow-up study with data collected via structured interviews between September 2019 and August 2020 in Feuang (rural) and Vangvieng (urban) districts in Vientiane province, Lao PDR. We identified and invited all women attending antenatal care in their third trimester of pregnancy in the selected areas. Using a structured questionnaire at third trimester of pregnancy we captured data on knowledge regarding antibiotic use and resistance. We collected information on attitudes and reported practice at two time points: (i) at third trimester of pregnancy and (ii) 6 months after birth. Univariate analysis and frequency distributions were used to study pattern of responses. Chi-square and Mann-Whitney tests were used to compare categorical and continuous variables respectively. *P* value < 0.05 was considered statistically significant.

**Results:**

We surveyed 539 women with a mean age of 25 years. Two oral antibiotics, i) ampicillin and ii) amoxicillin were correctly identified by 68 and 47% of participants respectively. Only 24% of women (19% in Feuang and 29% in Vangvieng) answered correctly that antibiotics are effective against bacterial infections. The most prevalent response was “I don’t know” suggesting the questions were challenging. Significantly less women would use antibiotics from a previous illness for their child than for themselves (16% vs 29%), however they would be more willing to use antibiotics for their baby even in case of mild symptoms (29% vs 17% while pregnant). The majority of antibiotics were prescribed by healthcare providers and 46% of children with the common cold received antibiotics.

**Conclusions:**

Women’s knowledge was sub-optimal, still, they manifested appropriate attitudes towards antibiotic use during pregnancy and for their child. Nearly half of children received antibiotics for the common cold. There is a need for context adapted programs aiming at improving women’s knowledge, as well as healthcare providers, emphasising rational antibiotic prescribing during pregnancy and for children.

## Introduction

Across various healthcare settings and contexts, antibiotics are among the medicines most commonly used during pregnancy to manage bacterial infections [1], to prevent maternal and neonatal morbidity and mortality [2, 3]. Antibiotic use during pregnancy spans from 20.8% in the Netherlands to 40.8% in the United States [1, 4] and urinary tract infections (UTIs) account for the majority of their use [4].

Antibiotics are frequently prescribed to children from the first days of their life. It was shown that more than 70% of newborns admitted to neonatal intensive care units receive antibiotics, with large variation across the world [5, 6]. Previous European studies have shown high prevalence of antibiotic prescribing for children under six [7] with the highest proportion of antibiotic prescriptions for two year old children (29%) in comparison to other age categories [8]. A study from rural Vietnam showed that 62% of children under five had been given antibiotics during a 28-day reporting period with the main reason given as acute respiratory tract infection [9].

Prescribing antibiotics in pregnancy raises a long-debated risk-benefit dilemma. It requires careful consideration due to a possible teratogenic effect on the developing foetus. Antibiotics influence the microbial environment of the mother and may impact the child already in utero, disrupting the correct establishment of the nascent microbiota and hence the microbial infant’s gut colonisation. Moreover, alternated vaginal microbiota can be a vehicle for a non-favourable transfer of microbes to the baby during birth [10]. These may lead to higher neonatal morbidity, and later in childhood higher susceptibility to infections and immune-mediated diseases [11, 12] and increased antibiotic consumption.

The administration of antibiotics early in life can contribute to a greater risk of childhood overweight, obesity and allergies, especially asthma [13–15]. Also, there is increasing evidence showing that antibiotic resistance is a significant issue in neonatal units across the world [16]. In a broader perspective, only in 2019, 1.27 million deaths globally were attributable to bacterial antimicrobial resistance and 254,000 deaths at the regional level in Southeast Asia, east Asia and Oceania [17].

Women are usually the main caregivers of their children and often the main decision-makers regarding healthcare seeking behaviour and play a pivotal role in children’s disease management. Therefore, their knowledge, attitude and practice towards antibiotics impact the use of antibiotics for their children thus are of paramount importance. In a study from Vietnam, only 13% of caregivers (among which 92% were women) demonstrated correct overall knowledge in accordance with standard guidelines [9]. A study from Nigeria demonstrated that only 51% of breastfeeding mothers believed that antibiotics are used to treat bacterial infections [18]. Other studies have shown that parents have inadequate knowledge regarding antibiotic use specifically for respiratory tract infections [19, 20]. A study by Haenssgen [21] in rural Thailand and Lao PDR showed high levels of villagers’ community awareness of antibiotics and antibiotic resistance and infrequent access to antibiotics from informal sources. The most reported reasons for antibiotic use were the treatment of external wounds, cough and fever. However this study presented a view of the general population which points towards knowlege gaps in our understanding of pregnant women knowledge, attitude and practice during pregnancy and after birth towards their newborn children.

The quality of antenatal care (ANC) services has improved in Lao PDR. Most women give birth at health facilities, where antibiotics have been shown to be widely administered during normal vaginal deliveries [22]. At the same time there is a paucity of data regarding women’s knowledge, attitudes, and practices regarding antibiotic use and resistance. The main objective of this study was to evaluate knowledge, attitude and reported practice of pregnant women regarding antibiotic use and antibiotic resistance during their pregnancy. Further, to evaluate the same parameters in regards to their newborn children. The study findings are essential to address the current knowledge gap and to design context specific interventions to improve rational antibiotic use (short-term) and contain antibiotic resistance (long-term).

## Methods

### Study design and participants

**Fig. 1:**
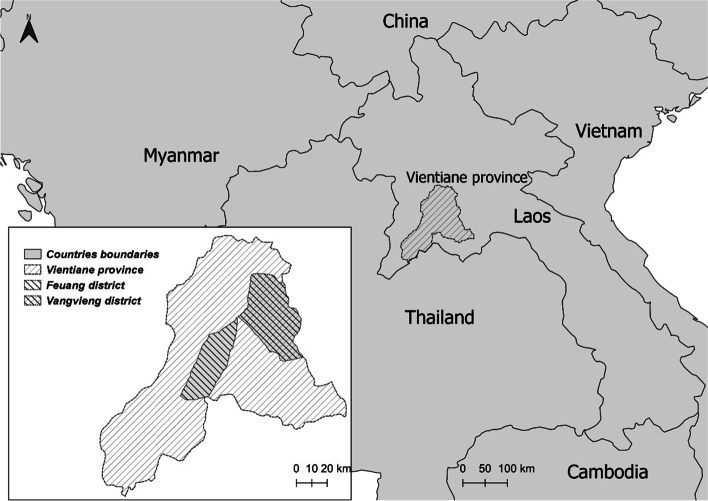
Map of Lao PDR and the study areas

This was a follow-up study conducted in Vientiane province, 60 km from Vientiane capital in Lao PDR between September 2019 and August 2020. Two districts, Feuang and Vangvieng were purposively selected as study areas based on variable background characteristics of the participants (e.g., rural vs urban population, different distribution of minority groups) and existing research area of Lao Tropical and Public Health Institute. The detailed description of the study setting and the calculation of sample size have been described in detail previously [23]. In Lao PDR, infectious disease morbidity and mortality is still high as well as neonatal mortality. The recent data showed that in Vientiane province alone, neonatal mortality rate was 20/1000 live births, infant mortality rate was 40/1000 live births, and under five mortality rate was 43/1000 live births [24]. Figure 1 represents the map of the study setting.

### Sampling process

**Fig. 2:**
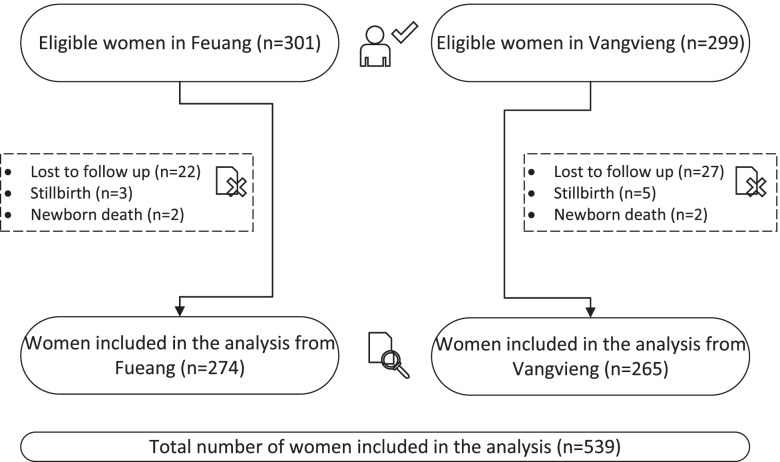
Study diagram

The local health authorities in each study district were approached by the research team and asked to identify all pregnant women who had at least one ANC visit in the district hospital and/or a health centre. The study inclusion criteria were as follows: (i) third trimester of pregnancy; (ii) residency in Feuang or Vangvieng districts (longer than six months at the time of recruitment), with no intention to move out in the following two years; (iii) voluntary agreement to join the study and be followed up until the child was 12 months. Women who had stillbirth or whose child died after delivery were not followed up. After obtaining permission from the local authorities, the purpose of the study was explained to all pregnant women by the researchers either at the health centre facility or at home. Written informed consent was obtained from all participants above 18 years old. Participants aged 15–17 years provided consent without guardian consent based on the assumption that young people of this age are competent (Gillick Competency Principle). This is consistent with Lao family law which recognises young people aged 15–17 are able to provide informed consent. In total 600 women (301 in Feuang and 299 in Vangvieng) were eligible for the study and gave informed consent at recruitment. At six months follow up, only 539 mothers were available for the interview and therefore included in the analysis. During the follow- up time, eight women had stillbirths (three in Feuang and five in Vangvieng) and four newborns died (two in each district). The 49 women who were lost to follow up (22 in Feuang and 27 in Vangvieng) moved outside the study area and could not be reached by telephone (Fig. 2).

### Development of data collection instruments

The structured questionnaires were developed based on previously used instruments by the study team and other researchers [25–27]. The questionnaires were adapted to collect data on knowledge, attitudes and reported practice towards antibiotic use and antibiotic resistance among study participants. The study instruments were pre-tested in Phonehong district (Vientiane province) and Sangthong district (Vientiane capital) with 30 women in their third trimester of pregnancy and 30 mothers of children under two years of age to check for clarity and comprehension of the questions. Based on the pre-test results, the questionnaires were adjusted. Subsequently, a three-day refresher training was provided to all data collectors. The final questionnaires consisted of five parts: (i) socio-demographic characteristics (age, level of education, occupation, ethnicity, family size, household income, ANC visits, number of pregnancies, and birth mode); (ii) reported antibiotic use practice during pregnancy (disease episodes or symptom during pregnancy, medicine consumption, health seeking practice, source of medicine); (iii) reported practice of giving antibiotics to a child (asked only if mother reported child sickness); (iv) knowledge about antibiotics consisted of seven questions to measure the capacity of recognising / identifying antibiotics from a matrix of medicines, effectiveness and side effects of antibiotic use, and nine questions of knowledge about antibiotic resistance; (v) 15 statements related to attitudes towards antibiotic use (e.g. demand antibiotics from healthcare providers). Knowledge of pregnant women was captured at third trimester of pregnancy by a set of questions on antibiotic effect and antibiotic resistance, and exercise pertaining their capacity of recognising / identifying antibiotics from a matrix of medicines. Women’s attitudes toward antibiotics were evaluated at two occasions; during their pregnancy and six months or later after their child was born. The questions asked were exactly the same with a difference that at the first occasion the questions were referring to themselves and at the second occasion were concerning their child.

### Data collection

Data were collected at two time points: 1) from September to November 2019 among women in their third semester of pregnancy; and 2) from March to August 2020 among the same women at six months after birth. All recruited pregnant women were informed about the purpose of the study and consented to participate. The interviews were performed at a convenient place either outside the ANC room or during a household visit. All the follow-up questionnaires with the women after birth were performed during the household visit. Data were captured digitally using tablets.

### Data management and statistical analysis

All questions were designed and coded in Research Electronic Data Capture (REDCap) software before field data collection [28]. Collected data were checked daily for consistency and completeness during field data collection and before uploading to the REDCap server. The data were analysed using STATA version 16 (College Station, Texas, USA). Univariate analysis and frequency distributions were used to study pattern of responses. Chi-square test was used to compare two categorical variables, and Mann–Whitney was used to compare continuous variables, particularly differences between Feuang (rural area) and Vangvieng district (urban area); a *p* value < 0.05 was considered statistically significant. The terms optimal/ sub-optimal were used to describe knowledge when more/less than 50% of correct answers were given. Appropriate/inappropriate attitudes were classified analogically. The assumption was based on previously published literature [29].

## Results

In total, 274 women from Feuang and 265 women from Vangvieng districts participated in the study. There were significant differences in general characteristics between the study participants in the two included areas. Compared to Feuang, women in Vangvieng were older (25.5 vs 23.9 years, *p* < 0.01), had higher level of education (*p* < 0.01), higher income (*p* < 0.01), which to less extent was earned in farming and more than half belonged to Lao-Tai, main ethnic group (*p* < 0.01). There were no significant differences in the number of pregnancies, children born or mode of previous birth between these two districts. The detailed characteristics of the study participants is presented in Table 1.

**Table 1:** General characteristics of participants in the two study districts

Characteristics	Total (***n*** = 539)	Feuang (***n*** = 274, 51%)	Vangvieng (***n*** = 265,49%)	***P-value***
Age (mean SD)	24.7 ± 6.1	23.9 ± 6.0	25.5 ± 6.0	< 0.01
**Household size** (median number of people, IQR)	5 (4–7)	6 (4–8)	5 (4–7)	0.10
**Income**^a^ (Lao Kips) (median, IQR)	2000,.000 (1000,000-3,000,000)	1,200,000 (600,000-2,500,000)	2000,000 (1,500,000-3,500,000)	< 0.01
**Ethnic-group**				< 0.01
Lao-Tai (main ethnic group)	247 (45.8)	106 (38.6)	141 (53.2)	
Other ethnic groups	292 (54.1)	168 (61.3)	124 (46.7)	
**Education**				< 0.01
Illiterate	61 (11.3)	36 (13.1)	25 (9.4)	
Primary	164 (30.4)	107 (39.0)	57 (21.5)	
Lower secondary and higher	314 (58.2)	131(47.8)	183 (69.0)	
**Occupation**				< 0.01
Farmer	254 (47.1)	147 (53.6)	107 (40.3)	
Housewife/work without pay	190 (35.2)	89 (32.4)	101 (38.1)	
Work with pay	95 (17.6)	38 (13.8)	57(21.5)	
**Number of pregnancies**				
1	181 (33.5)	88 (32.1)	93 (35.0)	0.27
2	126 (23.3)	59 (21.5)	67 (25.2)	
> =3	232 (43.0)	127 (46.4)	105 (39.6)	
**ANC visits during pregnancy**				0.04
< =1	33 (6.1)	14 (5.1)	19 (7.1)	
2–3	50 (9.2)	18 (6.5)	32 (12.0)	
> =4	456 (84.6)	242 (88.3)	214 (80.7)	
**Delivery mode**				0.37
Vaginal	512 (94.8)	258 (94.1)	254 (95.8)	
Assisted or c-section	27 (5.2)	16 (5.8)	11(4.1)	

*Abbreviations: ANC* antenatal care, *SD* standard deviation, *IQR* Interquartile range, ^a^ Income information was missing from 20 women

### Knowledge about antibiotics and antibiotic resistance

**Fig. 3:**
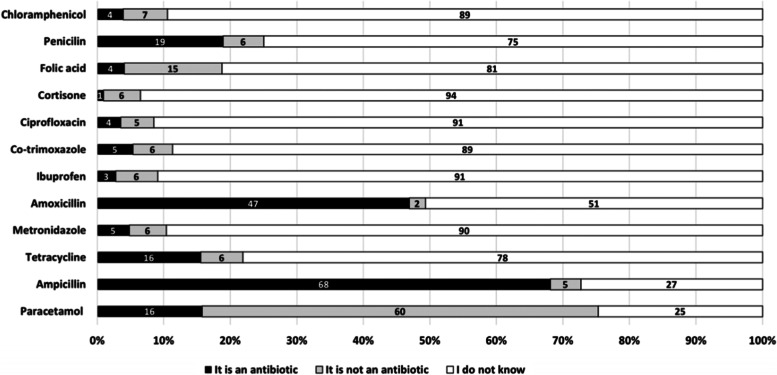
Identification of medicines by study participants in the two study districts

Among 13 pictures of the packages from the most common brands of medicines, only three medicines (i) paracetamol (not an antibiotic), (ii) ampicillin and (iii) amoxicillin (antibiotics) were identified correctly by nearly half or more of the participants (Fig. 3).

Only around one fifth of study participants mentioned that antibiotics are effective against bacterial infections and antibiotic use may cause side effects, while 47% of them declared to decide themselves on when to stop taking antibiotics. Only 16.5% of women heard the term antibiotic resistance (13.5% in Feuang and 19.6% in Vangvieng). Significantly more women were aware in Vangvieng (41%) than in Feuang (22%) that unnecessary use of antibiotics can make them ineffective in long term. However, it is noteworthy that in many questions the most prevalent response was “I don’t know” showing that the questions were challenging. Nearly 70% of women gave this answer when asked about antibiotic use in society and the risk of antibiotic resistance development and spread (Table 2).

**Table 2:** Knowledge of pregnant women about antibiotics in the two study districts

Knowledge Questions		Total (*n* = 539)	Feuang (*n* = 274, 50.8%)	Vangvieng (*n* = 265, 49.2%)	*P*
***Section 1. Knowledge about effectiveness of antibiotics***					
Q1. Antibiotics are effective against bacterial infections	Yes	129 (23.9)	52 (19.0)	77 (29.1)	< 0.01
	No	36 (6.7)	4 (1.5)	32 (12.1)	
	I don’t know	374 (69.4)	218 (79.6)	156 (58.9)	
Q2. Antibiotics are effective against viral infections	Yes	125 (23.2)	41 (15.0)	84 (31.7)	< 0.01
	No	39 (7.2)	13 (4.7)	26 (9.8)	
	I don’t know	375 (69.6)	220 (80.3)	155 (58.5)	
Q3. Common cold is normally caused by bacteria	Yes	96 (17.8)	34 (12.4)	62 (23.4)	< 0.01
	No	76 (14.1)	17 (6.2)	59 (22.3)	
	I don’t know	367 (68.1)	223 (81.4)	144 (54.3)	
Q4. Common cold is normally caused by viruses	Yes	127 (23.6)	39 (14.2)	88 (33.2)	< 0.01
	No	45 (8.4)	13 (4.7)	32 (12.1)	
	I don’t know	367 (68.1)	222 (81.0)	145 (54.7)	
Q5. Antibiotics are used to stop fever	Yes	163 (30.2)	86 (31.4)	77 (29.1)	< 0.01
	No	153 (28.4)	58 (21.3)	95 (35.9)	
	I don’t know	223 (41.4)	130 (47.5)	93 (35.1)	
Q6. Antibiotic use may cause side effects	Yes	131 (24.3)	43 (15.7)	88 (33.2)	< 0.01
	No	71 (13.2)	50 (18.3)	21 (7.9)	
	I don’t know	337 (62.5)	181 (66.1)	156 (58.9)	
Q7. I decide myself when to stop antibiotics	Yes	256 (47.5)	146 (53.3)	110 (41.5)	0.02
	No	169 (31.4)	77 (28.1)	92 (34.7)	
	I don’t know	114 (21.2)	51 (18.6)	63 (23.8)	
***Section 2. Knowledge about antibiotic resistance***					
Q1. Have you heard the term ABR	Yes	89 (16.5)	37 (13.5)	52 (19.6)	0.11
	No	348 (64.6)	187 (68.3)	161 (60.8)	
	I don’t know	102 (18.9)	50 (18.3)	52 (19.6)	
Q2. Antibiotic use can cause ABR	Yes	117 (21.7)	50 (18.3)	67 (25.3)	< 0.01
	No	72 (13.4)	29 (10.6)	43 (16.2)	
	I don’t know	350 (64.9)	195 (71.2)	155 (58.5)	
Q3. The more antibiotics we use in society, the higher risk that the resistance develops and spread	Yes	142 (26.4)	51 (18.6)	91 (34.3)	< 0.01
	No	38 (7.1)	17 (6.2)	21 (7.9)	
	I don’t know	359 (66.6)	206 (75.2)	153 (57.7)	
Q4. Bacteria can become resistant to antibiotics	Yes	85 (15.8)	32 (11.7)	53 (20.0)	< 0.01
	No	39 (7.24)	10 (3.7)	29 (10.9)	
	I don’t know	415 (77.0)	232 (84.7)	183 (69.1)	
Q5. People can become resistant to antibiotics	Yes	132 (24.5)	51 (18.8)	81 (30.6)	< 0.01
	No	70 (13.0)	39 (14.2)	31(11.7)	
	I don’t know	337 (62.5)	184 (67.2)	153 (57.7)	
Q6. Unnecessary use of antibiotics can make them ineffective in long term	Yes	170 (31.5)	61 (22.3)	109 (41.1)	< 0.01
	No	45 (8.4)	21 (7.7)	24 (9.1)	
	I don’t know	324 (60.1)	192 (70.1)	132 (49.8)	
Q7. ABR can spread from animals to humans	Yes	85 (15.8)	37 (13.5)	48 (18.1)	0.01
	No	80 (14.8)	31 (11.3)	49 (18.5)	
	I don’t know	374 (69.4)	206 (75.2)	168 (63.4)	
Q8. Today ABR is a big problem in Lao PDR	Yes	121 (22.5)	47 (17.2)	74 (27.9)	< 0.01
	No	48 (8.9)	13 (4.7)	35 (13.2)	
	don’t know	370 (68.7)	214 (78.1)	156 (58.9)	
Q9. Today, ABR is a big problem in the world	Yes	91 (16.9)	37 (13.5)	54 (20.4)	< 0.01
	No	45 (8.4)	8 (2.9)	37 (14.0)	
	don’t know	403 (74.8)	229 (83.6)	174 (65.7)	

*Abbreviations: ABR* antibiotic resistance

### Attitudes towards antibiotic use

The majority of women, when asked about their attitudes towards antibiotic use during pregnancy and for their child, agreed that the doctors conduct examinations (90 and 93%, respectively). If antibiotics are prescribed, the healthcare provider (doctor, nurse or pharmacist) would take time to provide sufficient information about the antibiotic use (93 and 96%, respectively). Also, women were confident about a doctor’s decision even in case of not prescribing an antibiotic. There were significant differences across several attitudes related questions between when women were pregnant and when they became mothers. In case of mild symptoms, 29% of mothers would use antibiotics for their child compared to 17% of pregnant women who would use them during pregnancy. In addition, mothers would expect antibiotics being prescribed by the doctor/ assistant doctor when their child suffers from a common cold to a higher extent compared to themselves during pregnancy (36% vs 26% respectively). On the other hand, 87% of mothers would not use leftover antibiotics for their newborn compared to 79% of pregnant women who would not use it during their pregnancy (Table 3).

**Table 3:** Attitudes towards antibiotic use of women during pregnancy and after birth for their children (six months follow up)

Attitude Questions	Woman during pregnancy	Woman after birth (six months follow up)	*p value*
Q1 When ill e.g., having a runny nose, sore throat, fever or cough but without difficulties in breathing I would use antibiotics to get better quickly			< 0.01
Disagree	411 (76.2)	366 (67.9)	
Agree	92 (17.0)	156 (28.9)	
I don’t know	36 (6.6)	17 (3.1)	
Q2. If one of my family members is ill with the above symptoms I would advise them to take antibiotics without seeing a healthcare provider			< 0.01
Disagree	282 (52.3)	341 (63.2)	
Agree	225 (41.7)	181 (33.5)	
I don’t know	32 (5.9)	17 (3.1)	
Q3. I expect antibiotics being prescribed by the doctor/ assistant doctor when suffering from a common cold			< 0.01
Disagree	341 (63.2)	316 (58.6)	
Agree	141 (26.1)	192 (35.6)	
I don’t know	57 (10.5)	31 (5.7)	
Q4. I prefer to be able to buy antibiotics directly from a pharmacy without prescription			< 0.01
Disagree	396 (73.4)	445 (82.5)	
Agree	119 (22.0)	84 (15.5)	
I don’t know	24 (4.4)	10 (1.8)	
Q5. If I would have left over antibiotics, I would prefer to store them at home, just in case I need them later			< 0.01
Disagree	384 (71.2)	452 (83.8)	
Agree	137 (25.4)	77 (14.2)	
I don’t know	18 (3.3)	10 (1.8)	
Q6. I would use leftover antibiotic from previous prescription for treating similar symptoms			< 0.01
Disagree	428 (79.4)	471 (87.5)	
Agree	90 (16.7)	59 (10.9)	
I don’t know	21 (3.9)	8 (1.4)	
Q7. When getting a cold I will use antibiotics to prevent symptoms from getting worse			< 0.01
Disagree	382 (70.8)	404 (74.9)	
Agree	116 (21.5)	124 (23.0)	
I don’t know	41 (7.6)	11 (2.0)	
Q8. I think it is good to be able to acquire antibiotics from relatives or acquaintances without seen by a healthcare provider			0.13
Disagree	458 (84.9)	480 (89.0)	
Agree	62 (11.5)	46 (8.5)	
I don’t know	19 (3.5)	13 (2.4)	
Q9. I would change the doctor/assistant doctor if according to my opinion they do not prescribe antibiotics enough			0.03
Disagree	402 (74.5)	428 (79.5)	
Agree	95 (17.6)	88 (16.3)	
I don’t know	42 (7.7)	22 (4.0)	
Q10. Doctor/assistant doctor always conduct an examination regardless if patient needs antibiotic or not			0.29
Disagree	41 (7.6)	32 (5.9)	
Agree	485 (89.9)	499 (92.5)	
I don’t know	13 (2.4)	8 (1.4)	
Q11. Doctor/assistant doctor prescribe antibiotics when patient expects it			< 0.01
Disagree	337 (62.5)	335 (62.1)	
Agree	161 (29.8)	192 (35.6)	
I don’t know	41 (7.6)	12 (2.2)	
Q12. When antibiotics are prescribed, the doctor/assistant doctor takes time to provide information on how they should be used, in an understandable manner			0.08
Disagree	28 (5.2)	18 (3.3)	
Agree	500 (92.9)	517 (95.9)	
I don’t know	10 (1.8)	4 (0.7)	
Q13. Pharmacy staff take their time to inform me on how antibiotics should be used			0.12
Disagree	27 (5.0)	14 (2.6)	
Agree	505 (93.8)	519 (96.2)	
I don’t know	6 (1.1)	6 (1.1)	
Q14. A doctor/assistant doctor who does not prescribe antibiotics when patient thinks that they are needed, is not a good doctor			< 0.01
Disagree	388 (71.9)	381 (70.6)	
Agree	102 (18.9)	135 (25.0)	
I don’t know	49 (9.0)	23 (4.2)	
Q15. I am confident in a doctor’s/assistant doctor’s decision if she/he does not prescribe antibiotics			< 0.01
Disagree	38 (7.0)	60 (11.1)	
Agree	480 (89.0)	471 (87.3)	
I don’t know	21 (3.9)	8 (1.4)	

### Reported practice of participants regarding antibiotic use during pregnancy and for their children

Only 18% of women (96/539) reported sickness during pregnancy: 73% of them (70/96) reported at least one episode, and 15% (14/96) two episodes. The most common symptoms/illness reported by pregnant women were the common cold (39%, 37/96) followed by fever (25%, 24/96), reproductive tract infections (abnormal discharge, pelvic pain, itching) (20%, 19/96), pneumonia (rapid and difficult breathing, chest pain, a severe cough that may produce phlegm, wheezing, fever and fatigue) (7%, 7/96), and UTIs (urinary urgency and/or burning) (5%, 5/96) whereas 4% (4/96) had other or combination of symptoms. Sixty percent (324/539) of women reported that their child got sick at least once and among them 73% (235/324) reported their child had one-two episodes and 27% reported their child had three (or more) episodes of diseases/symptoms (89/324). The most common symptoms/illness among children were the common cold (75%, 243/324), fever (22%, 72/324), pneumonia (6%, 19/324), and UTIs (5%, 16/324). Among the 37 women who reported the common cold, 62% (23/37) reported using medicines and 19% (7/23 antibiotics. On the other hand, for 243 children with reported the common cold, 94% (229/243) were given medicine and among them 46% (112/229) were given antibiotics. Almost all pregnant women (86%, 12/14) and mothers (98%, 143/146) reported that healthcare providers prescribed antibiotics for them and their child. Most of them purchased antibiotics from health facilities (pregnant women: 86%, 12/14; child: 81%, 118/146). Table 4 represents the reported practice of women taking antibiotics during pregnancy and antibiotic use for their child.

**Table 4:** Reported practice of taking antibiotics during pregnancy and using antibiotics for the children

		Most commonly reported symptoms/illness episodes				
		Common cold n (%)	Pneumonia n (%)	UTI n (%)	RTI n (%)	Fever n (%)
Pregnant women who reported symptoms/illness during pregnancy (*n* = 96)		37 (40.2)	7 (7.6)	5 (5.4)	19 (20.6)	24 (26.0)
Children with reported symptoms/illness (*n* = 324)		243 (75.5)	19 (5.8)	16 (4.9)	–	72 (22.2)
***Got medicine for this symptom***						
Pregnant women		23 (62.0)	1(14.0)	0	7(37.0)	20 (83.0)
Child		229 (94.0)	15 (79.0)	12 (75.0)	–	67 (93.0)
***Antibiotic used***						
Pregnant woman		7 (19.0)	0	–	5 (26.0)	2(8.0)
Child		112 (46.0)	15 (79.0)	8 (50.0)	–	11 (15.0)
***If antibiotic was used, who suggested it?***						
Pregnant woman	Self-decision	1 (14.3)	–	–	1 (20.0)	0
	Healthcare providers	6 (86.0)	–	–	4 (80.0)	2 (100.0)
Child	Self- decision	2 (2.0)	1 (7.0)	0	–	0
	Healthcare providers	110 (98.0)	14 (93.0)	8(100.0)	–	11(100.0)
***If antibiotics was used, where did you get it?***						
Pregnant woman	Private pharmacy or drug store	1 (14.0)	–	–	1(20.0)	0
	Health facilities	6 (86.0)	–	–	4 (80.0)	2 (100.0)
Child	Private pharmacy or drug store	21 (19.0)	1(7.0)	4 (50.0)	–	2 (18.0)
	Health facilities	91 (81.0)	14 (93.0)	4 (50.0)	–	9 (82.0)

*Abbreviations: UTI* urinary tract infection, *RTI* respiratory tract infection

## Discussion

To the best of our knowledge, this is the first study describing knowledge, attitudes and reported practice of pregnant women regarding antibiotic use during pregnancy and in early childhood in Lao PDR and other settings. The study revealed that knowledge of participants regarding antibiotics was sub-optimal. A limited number of women rightly identified antibiotics as medicines against bacteria. Overall women had appropriate attitudes towards antibiotic use during their pregnancy and were careful and considerate for their children. There were significant sociodemographic differences between the participants from urban and rural areas. We observed that a high proportion of antibiotics (86% during pregnancy and 98% for children) were used according to healthcare providers’ prescription that were obtained at health facilities. Antibiotics were broadly used for children with common cold during the first six months of their life.

Overall, the knowledge of study participants was sub-optimal and many participants tended to give ‘I don’t know’ answers which pointed towards difficulties in answering the questionnaire. The most well-known antibiotics among the study participants were ampicillin and amoxicillin, and only a few pregnant women recognised penicillin, chloramphenicol, metronidazole or co-trimoxazole as antibiotics. It should be noted that questions commonly used in questionnaires aiming to measure specific knowledge area (this study included) may not be aligned with the level of information that is available for the general public via common channels such as media, school education or information received from healthcare providers. We acknowledge that some questions are difficult to answer or might have been misunderstood by respondents. This is not only limited to the Lao PDR context. Several previous studies have investigated the caregivers’ knowledge about antibiotics [30, 31]. A study from Cyprus showed that most parents (64%) were able to identify correctly 60% of antibiotic names [31]. A study from northern Ethiopia conducted among 384 parents, showed that nearly half of them had poor knowledge regarding the use of antibiotics for children with upper respiratory tract infections-one of the most common indication for antibiotic prescribing [19]. Similarly, a study from Malaysia showed that parents often had inadequate knowledge and misconceptions about antibiotic use [20]. Moreover, some questions posed, seem to have no right or wrong answer considering the current state of knowledge, for example those evaluating the antibiotic resistance situation in Lao PDR. As reported in our recent study, the antibiotic resistance situation in Lao PDR is not as severe as in surrounding countries but is emerging quickly [32]. However, data are scattered what challenges a possibility of ascertained comparison between the countries for experts, and therefore cannot be expected from community members. Participants in our study were able to recognise most broad- spectrum antibiotics, namely ampicillin and amoxicillin which are commonly available and prescribed in these settings. A study conducted by Haenssgen et al., showed that respondents in Salavan province in Lao PDR were familiar with these antibiotics and were more likely to use various colloquial expressions to name them, like ‘Ampi’ with 76% and ‘Amok’ with 35% [21]. A previous study from Lao PDR showed that 83% of participants with reproductive tract infections/sexually transmitted infections symptoms used ampicillin (although not recommended for this indication) acquired mostly at local private pharmacies, which to a large extent were run by non-qualified staff [33]. Studies from other settings have demonstrated that aminopenicillins were a commonly prescribed antibiotic class. A study from the Netherlands showed that amoxicillin, followed by macrolides and amoxicillin/clavulanate were the most commonly prescribed antibiotics for infectious indications among children under two years old emphasising the practice of low prescribing for narrow-spectrum antibiotics [8].

We observed that women had appropriate attitudes towards antibiotic use during their pregnancy. However, a significant shift in attitudes was observed during several occasions regarding their children. It was evident that mothers tend to be more concerned about their born child compared to themselves during pregnancy. Their answers suggested that in case of sickness they were willing to help the newborns immediately. They reportedly would be more careful with using leftover antibiotics and were willing to follow the doctor’s advice and seek help at healthcare facilities for their children. This is in line with a European study conducted among parents (mostly mothers) where the majority (80%) accepted the clinician’s decision irrespective of antibiotics prescription [34]. However, our findings are in contrary to the previously published studies showing the preference for antibiotic self-treatment among the general population due to easy access without prescriptions from retail pharmacies and/or other informal sources [35–37]. Even though in Lao PDR self-medication with antibiotics is perceived prevalent, pregnant women were concerned about their own health during pregnancy and their newborn babies and the majority of them sought care at health care facilities where they received an antibiotic prescription directly from the healthcare provider. It is encouraging that the women in our study preferred to seek healthcare from formal structures and avoided self-medicating. Gender and access to health services may be one of the explanatory factors of the manifested health seeking behaviour. A study from Philippines showed that women due to being primary caregivers had greater knowledge of antibiotics, thus better health seeking behaviour as compared to fathers [38]. Also, parents who have good access to the health system consider themselves to be less anxious about their child’s health [31].

Other important findings in our study were significant differences in between the study districts which could possibly be explained by higher background education status among women in Vangvieng. Pregnant women in Feuang district were younger which is probably linked with a high number of early marriages in this area, where 61% of the population belongs to ethnic minority groups [39, 40]. As indicated previously in the Lao PDR Social Indicator Survey most women in the Lao-Tai ethnic group live in urban areas and have higher literacy as compared to other ethnic groups [24].

In the above-mentioned study from Cyprus residence area, ethnicity, parental age, sex and educational level were identified as significant factors associated with knowledge, attitudes and practices of parents concerning judicious antibiotic use for upper respiratory tract infections. Older parental age and higher educational level were clearly associated with better knowledge [31]. Importantly, in a recent study from Thailand gender together with socioeconomic status were presented as important determinants of seeking health information. Women with a higher level of education and socioeconomic status had 1.2 times higher odds of receiving public information about antibiotic use and resistance than men [41]. In a study from the UK, socioeconomic status has been shown as a determinant of antibiotic use during pregnancy where young women and women living in deprived areas were more likely to be prescribed antibiotics during pregnancy [4]. These led to a conclusion that efforts to improve antibiotic use and resistance should consider and adapt sociodemographic and cultural difference within the country.

However, one of the key findings was that antibiotics were commonly prescribed for the common cold for children during the first six months of their life. A drug utilisation study conducted in Lao PDR in 2006 in 30 health facilities showed that 97% of drugs received were per prescriptions and that 46% of children under five with simple diarrhoea received antibiotic prescription [42]. Another study from Lao PDR among 386 doctors attending morning meetings in 25 public hospitals showed that unnecessary antibiotic prescriptions were considered harmless by 30% participants, 60% declared not having enough information on antibiotic prescribing and 73% acknowledged issues with prescribing the correct antibiotics [43]. These results were in line with a recently published study by our team demonstrating that 36% of healthcare providers in Feuang and Vangvieng districts had below average knowledge regarding antibiotic use and resistance in both the rural and urban settings with 29% of healthcare providers stating that antibiotics are effective against viral infection [44]. This leads to a conclusion that there is an urgent need for interventions targeting healthcare providers focused on provision of local guidelines and recurrent educational activities.

### Strengths and limitations

The main strength of this study was a large sample size at enrolment and relatively low drop- out rate. Secondly, representativeness of women from both urban and rural setting was assured with strong focus on inclusion of the vulnerable ethnic minorities. Further, we collected data using Face-To-Face interviews by well-trained data collectors. As all other studies, this study had some limitations. The study districts were selected based on convenience sampling (i.e., existing research area of Lao Tropical and Public Health Institute) and therefore, may not be representative for the whole country. Antibiotic use during pregnancy and for the children was self-reported, therefore reporting and recall biases cannot be dismissed. Moreover, we did not verify if the reported episodes of symptoms/illness were confirmed by a healthcare provider. We can only assume that they were based on the fact that the majority of the medicines were used as a result of prescription in healthcare facilities.

## Conclusions and recommendations

In conclusion, this study demonstrated that pregnant women had sub-optimal knowledge about the use of antibiotics and antibiotic resistance, but showed appropriate attitudes and habits when it comes to using antibiotics for their newly born child. This emphasises the need for implementing context adapted programs aiming at increasing health literacy of pregnant women regarding antibiotic consumption and antibiotic resistance. However, the main attention should be focused on behaviour change interventions targeting healthcare providers and their prescribing practice. A significant part of the overall antibiotics used over the last two decades in Lao PDR has been shown unnecessary for common infectious indications.

Further research is needed to establish the optimal health literacy tool to study antibiotic consumption and antibiotic resistance among pregnant and breastfeeding women with an emphasis on children. Socioeconomic status and other sociodemographic characteristics must be considered when developing future programmes. Moreover, broader system-level initiatives should be established and strengthened to promote prudent antibiotics prescribing such as the implementation of the national action plan, prescribing guidelines, antibiotic stewardship programs for both out- and in-patient settings with a good surveillance and evaluation system in place.

## Data Availability

Relevant data for this study are presented in the tables. Any further data are available upon request from the corresponding author.
